# Serum uric acid level and its association with metabolic syndrome and carotid atherosclerosis in patients with type 2 diabetes

**DOI:** 10.1186/1475-2840-10-72

**Published:** 2011-08-04

**Authors:** Qin Li, Zhen Yang, Bin Lu, Jie Wen, Zi Ye, Lili Chen, Min He, Xiaoming Tao, Weiwei Zhang, Ying Huang, Zhaoyun Zhang, Shen Qu, Renming Hu

**Affiliations:** 1 Institute of endocrinology and diabetology, Huashan hospital, Shanghai medical college, Fudan University. 12 Middle Wulumuqi Road, Shanghai, China; 2 Department of endocrinology, Shanghai tenth people's hospital, School of medicine, Tongji University. 301 Middle Yanchang Road, Shanghai, China

**Keywords:** uric acid, metabolic syndrome, intima-media thickness, atherosclerosis

## Abstract

**Objective:**

We aimed to investigate whether elevated serum uric acid concentrations are associated with higher risk of metabolic syndrome (MetS) and carotid atherosclerosis in patients with type 2 diabetes.

**Methods:**

We conducted a population-based cross-sectional survey in Shanghai, with a total of 395 men and 631 women age 41 to 92 years. The carotid artery intima-media thickness (IMT) and carotid atherosclerotic plaques (PLQ) were measured by B-mode ultrasound. MetS was defined according to the updated National Cholesterol Education Program Adult Treatment Panel III criteria for Asian Americans.

**Results:**

Uric acid levels were negatively associated with duration of diabetes, fasting plasma glucose, glycohemoglobin, eGFR, HDL-cholesterol (all P < 0.001) and positively with BMI, CRP, waist circumference, triglycerides, systolic blood pressure, ACR, HOMA-IR and IMT (all P < 0.05). In the highest quartile of uric acid levels, the risks were substantially higher for MetS [odds ratio 3.97, (95% confidence interval 2.58-6.13)] (P < 0.001 for trend) and PLQ [odds ratio 2.71 (95% confidence interval 1.62-4.47)] (p = 0.013 for trend) compared with that in the lowest quartile of uric acid levels after multiple adjustment. These associations remained significant after further adjustment for potential confounders.

**Conclusions:**

Serum uric acid level is associated with MetS and is an independent risk factor for carotid atherosclerosis in patients with type 2 diabetes.

## Introduction

Metabolic syndrome (MetS), a clustering of cardiovascular risk factors such as insulin resistance, hypertension, glucose intolerance, hypertriglyceridemia, and low high-density lipoprotein (HDL) cholesterol levels, is a major worldwide public health problem[1]. The prevalence of MetS is increasing in China because of the westernization of the lifestyle, such as a high-fat and high-calorie diet and less physical activity. A recent epidemiological study indicated that the prevalence of MetS among Chinese men and women aged 35 to 64 years was 9.8% and 17.8%, respectively[2].

Uric acid is the end product of purine metabolism in humans. Excess serum accumulation can lead to various diseases, and most notably uric acid is causally involved in the pathogenesis of gouty arthritis [3-5]. Recent studies have suggested that hyperuricemia is a risk factor for cardiovascular disease (CVD) in the general population [6-8]. However, despite the clinical and epidemiological evidence, some authorities have considered that the association was confounded by other well-established risk factors for CVD [3,9].

Recently, growing evidence demonstrates that uric acid may have a key role in the pathogenesis of MetS [10]. Recent studies in animals models report that uric acid may play a causal role in the development of MetS and decreasing uric acid levels can prevent or reverse features of the MetS [11].

Although previous studies have analyzed the independence of the relationship between uric acid and MetS and carotid atherosclerosis, thus far, few have examined in type 2 diabetes and there is a scarcity of data evaluating the effect of MetS on the association between serum uric acid and carotid atherosclerosis. In this study, we aimed to investigate the association between the serum uric acid and MetS and carotid atherosclerosis in patients with type 2 diabetes.

## Methods

### Study subjects

A cross-sectional study to evaluate the prevalence of diabetic complications in Chinese patients aged over 30 and diagnosed with type 2 diabetes was planned in downtown Shanghai. The details of the study design have been described previously[12]. In brief, the study was conducted from February to July 2004. 20 residential areas administered by 20 residents' committees were sampled randomly in the central area of Shanghai. Questionnaires to identify diabetes history were sent to every household in the 20 residential areas and collected by primary care clinicians and endocrinologists. A total of 1120 Chinese patients diagnosed with type 2 diabetes were identified via the questionnaire. Finally, 1039 Chinese patients diagnosed with type 2 diabetes were enrolled in our study. After excluding those who did not have serum uric acid data (n = 13), 1026 individuals (395 men and 631 women) were eligible for the present analysis. The study was approved by the Institution Review Board of the Huashan Hospital, and written informed consents were obtained from all participants.

### Laboratory measurements

Overnight fasting blood samples were collected in tubes containing liquid EDTA, centrifuged at 4°C, and stored at -80°C until analysis. The measurements of total cholesterol, HDL-cholesterol, low-density lipoprotein (LDL) cholesterol, triglycerides, glucose, insulin, creatinine, and C-reactive protein (CRP) were previously described[12,13]. Insulin resistance was estimated using homeostasis model assessment index-insulin resistance (HOMA-IR) [14]. A sterile, random-spot urine sample was used to measure the albumin/creatinine ratio (ACR). We used the modification of diet in renal disease (MDRD) equation re-calibrated for Chinese to estimate eGFR expressed in ml/min/1.73 m^2^: eGFR = 186 × [SCR × 0.011]^-1.154 ^× [age]^-0.203 ^× [0.742 if female] × 1.233, where SCR is serum creatinine expressed as μmol/l and 1.233 is the coefficient for Chinese.

### Carotid ultrasonography

Carotid ultrasonography was performed using an Acuson Sequoia 512. Trained and certified sonographers conducted the examination. The ultrasound scanning protocol in our study was modified in terms of procedures used in previous studies [15-17]. Computer-assisted edge-detection software was not used for measurement of carotid intima-media thickness (IMT). A lateral view of bilateral images of common carotid arteries (1 cm proximal to the dilatation of the carotid bulb), carotid bulb (identified by the loss of the parallel wall present in the common carotid artery) and internal carotid artery (1 cm distal to the tip of the flow divider that separates the external and internal carotid arteries) was obtained. Sonographers recorded the images and completed ultrasound readings. IMT is the distance between the lumen-intima interface and the media-adventitia interface [18]. Common carotid artery IMT was defined as the mean of the maximum IMT in both right and left sides of common carotid artery.

The plaque of carotid artery (common carotid artery, carotid bulb and internal carotid artery) is defined as a localized protrusion of the internal part of the vessel wall into the lumen of 50% of the surrounding IMT value. Plaque presence was defined as ≥1 plaque in any of the carotid arteries [19].

### Definition of MetS

The MetS was defined based on the updated National Cholesterol Education Program Adult Treatment Panel III criteria for Asian-Americans[20]: (1) as presenting at least three of the following components: 1) waist circumferences 90 cm or greater in men or 80 cm or greater in women; 2) triglycerides 1.7 mmol/liter or greater; 3) HDL cholesterol less than 1.03 mmol/liter in men or less than 1.30 mmol/liter in women; 4) blood pressure 130/85 mm Hg or greater or current use of antihypertensive medications; or 5) fasting plasma glucose (FPG) 5.6 mmol/liter or greater or previously diagnosed type 2 diabetes or on oral antidiabetic agents or insulin.

### Statistical analysis

Normally distributed data were expressed as means ± SD, whereas variables with a skewed distribution were reported as median (interquartile range) and log transformed to approximate normality before analysis. Categorical variables were represented by frequency and percentage. Analysis of covariance for continuous variables and multivariate logistic regression analysis for categorical variables were applied for the comparison according to uric acid quartiles. Analysis of covariance was used to compare uric acid levels between genders. Correlation coefficients between uric acid and metabolic features were calculated by partial correlation analysis on ranks (Spearman correlation). Serum uric acid levels were depicted according to the number of MetS components using linear regression model. Multivariate logistic regression models were used to estimate the odds ratios (ORs) for MetS. Potential confounding variables including age, gender, smoking, alcohol drinking, self-reported CVD, family history of diabetes, eGFR, HbA_1C_, CRP, HOMA-IR and body mass index (BMI) were controlled in the regression models. Data management and statistical analysis were performed using SPSS version 13.0 for Windows (SPSS, Chicago, IL, USA). P < 0.05 was considered statistically significant.

## Results

### Characteristics of participants according to serum uric acid quartiles

We identified 1026 patients with type 2 diabetes with a mean age of 65.57 ± 11.70 years. At the time of uric acid determination, history of hypertension was documented in 761 patients (74.2%) and CVD in 200 patients (19.5%). Baseline demographic and medical characteristics for both genders combined and divided by uric acid quartiles are illustrated in Table 1. When analyzed by quartiles of uric acid levels, the patients with higher uric acid were more likely to be male and smokers (both P < 0.05). With respect to metabolic parameters, the patients in the higher uric acid quartiles exhibited higher levels of systolic blood pressure, BMI, waist circumference, HOMA-IR, CRP, IMT, creatinine, ACR and triglycerides (all P < 0.05). In contrast, the patients with higher uric acid levels displayed shorter duration of diabetes and lower levels of FPG, HbA_1C_, eGFR and HDL cholesterol (all P < 0.001).

**Table 1:** Characteristics of study participants according to uric acid quartiles^a^

Characteristics	Q1 (n = 256) <230	Q2 (n = 257) 231-280	Q3 (n = 256) 281-330	Q4 (n = 257) ≥331	*P *value
MetS (%)	20.3	35.8	54.3	84.8	<0.001

Uric acid (μmol/L)	200 (190-220)	260 (250-270)	310 (300-320)	390 (350-430)	<0.001
Age (yr)^b^	65.28 ± 11.27	66.57 ± 10.28	65.09 ± 12.35	67.52 ± 11.84	0.065
Male^b^	73 (28.29)	91 (30.33)	92 (41.81)	139 (56.05)	<0.001
Smoking (yes)	47 (18.22)	47 (15.67)	52 (23.64)	63 (25.40)	0.017
Alcohol (yes)	27 (10.47)	27 (9.00)	31 (15.50)	38 (15.32)	0.084
Duration of diabetes (yr)	9.72 ± 7.84	7.74 ± 7.04	7.24 ± 6.69	6.75 ± 6.56	<0.001
Self-reported CVD^c^	49 (18.99)	62 (20.67)	49 (22.27)	59 (23.79)	0.587
Family history of diabetes^d^	112 (43.41)	119 (39.67)	74 (33.64)	84 (33.87)	0.068
SBP (mmHg)	138.44 ± 20.68	139.92 ± 19.82	140.86 ± 22.00	142.31 ± 20.05	<0.001
DBP (mmHg)	80.71 ± 10.54	82.57 ± 10.92	82.14 ± 12.18	82.38 ± 10.69	0.207
BMI (kg/m^2^)	24.21 ± 3.39	24.91 ± 3.23	25.40 ± 3.47	25.97 ± 3.39	<0.001
Waist circumference (cm)	81.74 ± 9.68	83.62 ± 8.50	86.05 ± 9.32	87.67 ± 8.88	<0.001
Waist to hip ratio	0.86 ± 0.08	0.87 ± 0.06	0.88 ± 0.06	0.89 ± 0.06	0.176
Glucose (mmol/L)	9.25(7.20-12.30)	7.70(6.40-9.60)	7.80(6.60-9.70)	7.30(6.23-8.80)	<0.001
Insulin (μU/mL)^e^	9.93 (5.85-15.06)	11.06 (6.93-17.26)	12.40 (7.93-18.24)	13.19 (8.80-20.13)	0.143
HOMA-IR^e^	3.91 (2.28-6.70)	3.98 (2.36-7.21)	4.40 (2.62-7.38))	4.44 (2.63-6.55	0.028
CRP	1.92 (0.95-4.35)	1.98 (0.71-4.92)	2.33 (0.73-5.24)	3.59 (0.81-8.87)	<0.001
IMT (mm)	0.82 ± 0.21	0.85 ± 0.22	0.86 ± 0.26	0.93 ± 0.29	0.016
HbA_1C _(%)	7.91 ± 1.94	7.10 ± 1.59	6.96 ± 1.42	6.93 ± 1.36	<0.001
Creatinine (μmol/L)	60.67 ± 13.14	65.22 ± 19.63	67.35 ± 15.50	81.09 ± 28.75	<0.001
eGFR (ml/min/1.73^2^)	132.5 ± 25.5	125.2 ± 25.4	122.5 ± 26.7	108.3 ± 31.2	<0.001
ACR (mg/mmol)	2.45 (1.25-6.04)	2.80 (1.35-9.59)	3.02 (1.54-8.11)	3.52 (1.74-8.94)	0.022
Triglycerides (mmol/L)^e^	1.41 (0.97-1.91)	1.56 (1.09-2.23)	1.67 (1.17-2.41)	1.84 (1.28-2.64)	<0.001
Total cholerterol (mmol/L)	5.32 ± 0.07	5.37 ± 0.07	5.44 ± 0.08	5.33 ± 0.07	0.623
LDL cholesterol (mmol/L)	2.99 ± 0.90	3.04 ± 0.84	3.11 ± 0.86	3.07 ± 0.85	0.189
HDL cholesterol (mmol/L)	1.47 ± 0.40	1.37 ± 0.36	1.26 ± 0.35	1.14 ± 0.29	<0.001

SBP, systolic blood pressure; DBP, diastolic blood pressure.; IMT, intima-media thickness

^a^Data are means SD, median (interquartile range), or number (percent); P value was calculated after adjustment for age, gender.

^b^Not adjusted for itself.

^c^Self-reported CVD including stroke and coronary heart disease.

^d^Parents or siblings had a history of diabetes.

^e^These variables were log transformed before analysis.

### Association between serum uric acid and MetS

Partial correlation analysis demonstrated strong correlation between uric acid and BMI, waist circumference, HOMA-IR, CRP, TG and HDL cholesterol among various metabolic features (Table 2).

**Table 2:** Correlation between serum uric acid and other parameters in patients with type 2 diabetes^a^

Variable	Correlation coefficient	P value
Age	0.04	0.19
BMI	0.15	<0.001
Duration of diabetes	-0.14	<0.001
Waist circumference	0.21	<0.001
Waist to hip ratio	0.08	0.01
SBP	0.04	0.23
DBP	0.02	0.48
CRP	0.08	0.01
Glucose	-0.26	<0.001
Insulin	0.11	<0.001
HbA_1C_	-0.24	<0.001
HOMA-IR	0.07	0.02
IMT	0.07	0.02
Creatinine	0.33	<0.001
eGFR	-0.36	<0.001
ACR	0.11	<0.001
Triglycerides	-0.04	0.20
Total cholesterol	0.20	<0.001
LDL cholesterol	0.01	0.80
HDL cholesterol	-0.33	<0.001

IMT, intima-media thickness; SBP, systolic blood pressure; DBP, diastolic blood pressure;

^a^All correlation coefficients were calculated after adjustment for age, gender.

**Figure 1:**
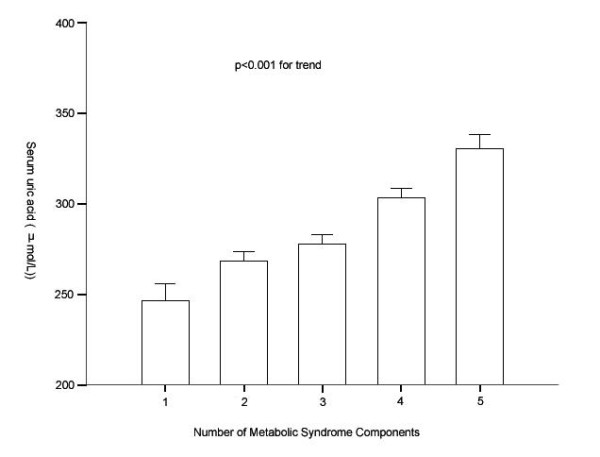
**Plasma uric acid levels according to the number of MetS components**. Data are shown as means ± SE after adjustment for age, gender, eGFR, alcohol drinking, smoking, duration of diabetes, self-reported CVD, and family history of diabetes; P < 0.001 for trend.

Remarkably, serum uric acid levels increased gradually with increasing number of MetS components (Figure 1). The mean values (SE) of uric acid concentrations significantly increased for those with one to five components were 246.67 (7.89), 268.31 (4.37), 277.91 (4.28), 303.35 (4.56), and 330.35 (6.70) μmol/L, respectively, after adjustment for age, gender, creatinine, alcohol drinking, smoking, duration of diabetes, self-reported CVD and family history of diabetes.

As presented in Table 3, the ORs for MetS were higher with increasing uric acid quartiles (P < 0.001 for trend). In the highest uric acid quartile, the ORs were 3.97 [95% confidence interval (CI) 2.58-6.13] for MetS after adjusting for age, gender, creatinine, alcohol drinking, smoking, duration of diabetes and family history of diabetes.

**Table 3:** Adjusted ORs and 95% CIs for MetS and carotid plaque according to uric acid quartiles

	ORs (95% CI)				P value for trend
		
	Q1	Q2	Q3	Q4	
MetS					
Model 1^a^	1.0	1.42 (0.99-2.04)	2.27 (1.51-3.42)	3.97 (2.58-6.13)	<0.001
Model 2^b^	1.0	1.37 (0.95-1.98)	2.21 (1.44-3.39)	3.65 (2.33-5.71)	<0.001
Model 3^c^	1.0	1.33 (0.88-2.07)	2.27 (1.36-3.71)	3.77 (2.26-6.29)	<0.001
Model 4^d^	1.0	1.22 (0.82-1.83)	1.93 (1.22-3.03)	3.26 (2.03-5.25)	<0.001
carotid plaque					
Model 1*	1.0	1.33 (0.88-1.89)	1.89 (1.16-2.85)	2.71 (1.62-4.47)	0.013
Model 2^†^	1.0	1.29 (0.82-1.81)	1.82 (1.13-2.77)	2.53 (1.49-4.13)	0.019
Model 3^‡^	1.0	1.22 (0.81-1.85)	1.68 (1.12-2.58)	2.42 (1.40-3.75)	0.020
Model 4^ξ^	1.0	1.18 (0.80-1.76)	1.52 (1.04-2.50)	2.21 (1.32-3.49)	0.036

^a^Model 1 adjusted for age and gender.

^b^Model 2 further adjusted for alcohol drinking, smoking, duration of diabetes, and family history of diabetes.

^c^Model 3 further adjusted for eGFR, CRP, HbA_1C _and HOMA-IR.

^d^Model 4 further adjusted for BMI.

*Model 1 adjusted for age and gender.

^†^Model 2 further adjusted for alcohol drinking, smoking, duration of diabetes, and family history of diabetes.

^‡^Model 3 further adjusted for eGFR, CRP, HbA_1C _and HOMA-IR.

^ξ^Model 4 further adjusted for BMI and MetS.

### Association between serum uric acid and carotid atherosclerosis

Partial correlation analysis demonstrated a significant correlation between uric acid and IMT (p = 0.02) (Table 2).

As presented in Table 3, the ORs for carotid atherosclerotic plaques (PLQ) were higher with increasing uric acid quartiles (p = 0.013 for trend). In the highest uric acid quartile, the ORs were 2.71 [95% confidence interval (CI) 1.62-4.47] for PLQ after adjusting for age, gender, eGFR, alcohol drinking, smoking, duration of diabetes and family history of diabetes.

### Effect of MetS on the association between serum uric acid and PLQ

After adjusting for age, gender, eGFR, alcohol drinking, smoking, duration of diabetes and family history of diabetes, MetS was associated with the PLQ with an odds ratio of 2.24 (95% CI 1.34-3.57, P < 0.001). Thus, we next investigated the association between serum uric acid and PLQ according to the status of MetS. After adjusting for age, gender, HbA_1C_, BMI, systolic blood pressure, diastolic blood pressure, FPG, alcohol drinking, smoking, duration of diabetes and family history of diabetes, in the highest uric acid quartile, the ORs were 1.85 (95% CI 1.14-2.83, p = 0.023) for carotid plaque in subjects without MetS and 3.07 (95% CI 1.25-6.42) in subjects with MetS.

### Serum uric acid level and glucose control

Hyperglycaemia as reflected by mean HbA_1C _levels was associated with lower serum uric acid levels. As summarized in Table 1, the patients with higher uric acid levels displayed lower HbA_1C _and fasting glucose levels (both P < 0.001). HbA_1C _and FPG were negatively correlated with serum uric acid (Table 2). Moreover, we also observed that serum uric acid levels were negatively related to the duration of diabetes (P < 0.001).

## Discussion

In the present study, we found that serum uric acid levels showed association with the risk of MetS and carotid atherosclerosis from the cross-sectional data in type 2 diabetes. Moreover, these associations are independent of lifestyle factors, eGFR, duration of diabetes, family history of diabetes and remarkably, CRP, HbA_1C_, HOMA-IR and BMI.

Possible associations between uric acid and MetS were evaluated in some studies [10,21]. We also observed that the ORs were substantially higher for MetS [OR 3.97, (95% confidence interval 2.58-6.13)] (P < 0.001 for trend) and PLQ [OR 2.71 (95% confidence interval 1.62-4.47)] (p = 0.013 for trend) in the highest serum uric acid quartile compared with those in the lowest quartile. In addition, we observed the serum uric acid concentration increased monotomically with the number of MetS components (P < 0.001 for trend). However, the underlying mechanisms of these associations are still largely unknown although much research has been done in this area. Recent studies indicate that hyperuricemia may be partially responsible for the proinflammatory endocrine imbalance in the vascular smooth muscle cells and adipose tissue, which is an underlying mechanism of the low-grade inflammation and insulin resistance in subjects with the CVD and MetS [22,23]. Lowering uric acid in mice by allopurinol could improve the proinflammatory endocrine imbalance in the adipose tissue by reducing production of monocyte chemoattractant protein-1 (MCP-1) and increasing production of adiponectin. In addition, lowering uric acid in obese mice decreased macrophage infiltration in the adipose tissue and reduced insulin resistance [21]. Moreover, uric acid has also been shown to induce production of interleukin 1β, interleukin 6, tumor necrosis factor α in human mononuclear cells, and CRP in cultured human vascular cells[24], all these have been suggested that uric acid may have some interaction with other inflammatory cytokines. In line with views, we observed significant correlation between serum uric acid and CRP levels even after adjustment for other potential confounders (r = 0.08, p = 0.01). Furthermore, we also observed that CRP increased with serum uric acid quartiles in this study (P < 0.001), which indicate that uric acid is correlated with low-grade systemic inflammatory response and could potentially modulate chronic inflammatory processes[25]. It is well-known that subclinical inflammation is a risk factor not only for MetS but also for diabetes complications such as CVD and stroke [26,27]. Taken together, these important and intriguing results suggest that uric acid may play a causal role in the pathogenesis of atherosclerosis and the MetS partially by inflammatory pathway.

Given the serum uric acid levels were significantly associated with atherosclerosis and the MetS[28,29], it is plausible to consider uric acid as a promising candidate for risk assessment and a potential intervention target for CVD and MetS. Interestingly, in line with our hypothesis, some studies have demonstrated that reducing uric acid by treatment with allopurinol significantly improved endothelial function, peripheral vasodilator capacity and blood flow both locally and systemically, which represent a new potential therapeutic approach [30-32]. Certainly, prospective studies with solid clinical end points are urgently needed to clarify whether a high uric acid level plays a causal role in the development of CVD and MetS.

One of the interesting findings of the present study is the inverse association of serum uric acid with diabetic parameters (duration of diabetes, HbA_1C_, FPG). The inverse association of serum uric acid with diabetic parameters is counterintuitive. Historically, the elevated level of uric acid observed in the MetS has been attributed to hyperinsulinemia, since hyperinsulinemia is known to be associated with impaired renal uric acid excretion and hyperuricemia in these patients are likely to be due to retention of uric acid[33]. But here in our current study, the type 2 diabetes patients with a higher HbA_1C_, fasting glucose and a longer duration of diabetes are likely to be accompanied by worsening function of beta cells to secrete adequate amounts of insulin. And the inverse relationship between serum uric acid and diabetic parameters (duration of diabetes, HbA_1C_, FPG) observed in these diabetic subjects may probably be caused by the increased renal excretion of uric acid in the presence of hyperglycaemia. It is well-known that with the increasing of the duration of diabetes, which is accompanied by worsening of the function of beta cells and deterioration of glycemic control, the rate of renal filtration in patients with diabetes increases gradually. The hyperfiltration state which caused by hyperglycaemia promotes the excretion of uric acid, which partly explain the existence of the inverse relationship between serum uric acid and diabetic parameters. Patients with long-term hyperfiltration state may eventually develop into diabetic nephropathy. Consistent with our hypothesis, we observed that ACR increased and eGFR decreased with serum uric acid quartiles in this study (p values were 0.022 and 3.7 × 10^-5^, respectively).

As a cross-sectional study, there are several limitations. The mechanisms underlying these associations are still to be explored. The present findings are inherently limited in the ability to eliminate causal relationships between uric acid and MetS. Although most potential confounders were carefully controlled, since some of the study patients had several risk factors including hypertension and dyslipidemia, we could not eliminate the possible effect of underlying diseases and medications used for these diseases on the present findings. Further prospective population-based trials are needed to investigate the mechanisms in order to answer these questions.

In conclusion, our data indicate that serum uric acid levels are significantly associated with MetS and carotid atherosclerosis in patients with type 2 diabetes, even after adjustment for other potential confounders. Furthermore, our findings suggest that the observed association between uric acid and carotid atherosclerosis may be attributed to metabolic syndrome-dependent and -independent mechanisms. Prospective studies are required to assess the time course and causal relevance of serum uric acid in the development of MetS and carotid atherosclerosis in patients with type 2 diabetes.

## Conflict of interests statement

The authors declare that they have no competing interests.

## Authors' contributions

ZY and RH designed the study; QL, ZY, LC, XT and WZ participated in acquisition of data; JW, ZZ, BL and SQ researched and evaluated the literature; ZY undertook the statistical analysis and QL wrote the first draft of the manuscript. All authors have approved the final manuscript for publication.
